# Functional and spatial segregation within the inferior frontal and superior temporal cortices during listening, articulation imagery, and production of vowels

**DOI:** 10.1038/s41598-017-17314-0

**Published:** 2017-12-05

**Authors:** Alessandra Cecilia Rampinini, Giacomo Handjaras, Andrea Leo, Luca Cecchetti, Emiliano Ricciardi, Giovanna Marotta, Pietro Pietrini

**Affiliations:** 1 IMT School for Advanced Studies, Lucca, 55100 Italy; 2 Department of Philology, Literature and Linguistics, University of Pisa, Pisa, 56100 Italy

## Abstract

Classical models of language localize speech perception in the left superior temporal and production in the inferior frontal cortex. Nonetheless, neuropsychological, structural and functional studies have questioned such subdivision, suggesting an interwoven organization of the speech function within these cortices. We tested whether sub-regions within frontal and temporal speech-related areas retain specific phonological representations during *both* perception and production. Using functional magnetic resonance imaging and multivoxel pattern analysis, we showed functional and spatial segregation across the left fronto-temporal cortex during listening, imagery and production of vowels. In accordance with classical models of language and evidence from functional studies, the inferior frontal and superior temporal cortices discriminated among perceived and produced vowels respectively, also engaging in the non-classical, alternative function – i.e. perception in the inferior frontal and production in the superior temporal cortex. Crucially, though, contiguous and non-overlapping sub-regions within these hubs performed either the classical or non-classical function, the latter also representing non-linguistic sounds (i.e., pure tones). Extending previous results and in line with integration theories, our findings not only demonstrate that sensitivity to speech listening exists in production-related regions and *vice versa*, but they also suggest that the nature of such interwoven organisation is built upon low-level perception.

## Introduction

According to classical models of speech processing, superior temporal and inferior frontal brain regions are consistently involved in perception and production, respectively^1^. However, theories dealing with the relationship between perceived and produced speech have long debated whether and to what extent perceptual and articulatory information are integrated in language acquisition and use, either assuming that perception shapes production, or that production influences perception^2,3^. Other proposals have instead suggested that articulatory coherence and perceptual value *both* contribute to a synergic processing of speech in the brain^4^.

The phoneme-specific specialization of the superior temporal cortex in perception, as well as that of a wide, prefrontal territory around Broca’s area in production, are well-known, since quite a few seminal studies have explored the neural encoding of phonological competence^5–8^. Interestingly, while the phoneme itself was a theoretical model debated mostly in Linguistics in the last century, many recent studies revealed that brain activity specific to phonological stimuli could be indeed isolated in the classical foci pertaining to perception and production, with both functional neuroimaging and electrophysiology methods^9^: particularly, the superior temporal cortex has been shown to represent the overall acoustic form of syllables^10^, syllable-embedded perceived consonants or vowel categories^11^, and even tones when phonologically marked^12^, while a precise account of motor involvement during production or imagery of phonemes has received less attention in the existing literature^13^.

Such rich and mixed picture sparked other questions: do distinct brain regions whatsoever support different aspects of speech processing (such as perception, imagery and production of phonemes)? Do they share specific phonological representations? In the context of theories debating an interwoven organization of speech perception and production, the Motor Theory of Speech Perception (MTSP)^3^ has argued in favour of a covert articulatory rehearsal mechanism, which would take place implicitly and automatically whenever a speaker is exposed to language, thus connecting the two ends of the perception-production continuum. Such mechanism was substantiated by findings generalized to other processes, crucially including motor control^14^.

In this respect, functional neuroimaging and electrophysiological studies have recently sought to determine the relationship between the perceptual and articulatory stages of speech, seeking perception-related information in frontal areas engaged by production tasks, and production-related information in temporal areas engaged by perception tasks^15–20^. In these studies, multivariate analyses were exploited to reveal similarities in informational content between regions previously inferred to perform different functions (through classical activation experiments), thus revealing a mixed picture of shared information and cortical space as well, and tangentially supporting integration models such as those described.

Similarly, virtual^21^ and real lesion studies failed to validate an exact correspondence between language impairments and information represented in the frontotemporal speech network: damage in one area may, or may not, entail loss of function in the other, as even sub-regions within such well-known perimeters appear to support different functions^22–25^. The idea of an interwoven cortical organization of speech function is also favoured by structural studies that reveal a fine-grained cytoarchitectonic, connectivity- and receptor-mapping-based parcellation of fronto-temporal language areas^26–31^. Therefore, disentangling the nature of the perception-production interface appears far from straightforward.

According to these indications, we tested whether sub-regions within the frontal and temporal speech areas retain specific, functionally segregated phonological representations during both perception and production, and whether a possible covert rehearsal mechanism could be elicited, through articulation imagery, to simulate the production-perception interface postulated by the MTSP (in contrast with hearing imagery^32,33^).

To this aim, using functional Magnetic Resonance Imaging (fMRI) and multivoxel pattern analysis (MVPA), we measured the spatial overlap of brain regions involved in stimulus-specific representations during vowel perception (listening), and production (imagined and overt articulation). Within a set of phonemes, the basic units of words, we selected vowels since they retain acoustic features (i.e., formants) that can combine together, thus to distinguish them in a discrete manner. Moreover, formant combinations emerge from unique articulatory gestures, so that their processing depends upon the same perceptuo-motor model^34^, differently from consonants^5,6,20^. Particularly, while consonants need to be embedded in syllables to be fully heard and articulated, vowels are self-standing phonemes with high salience. Vowels act as syllabic nuclei, prosodic aggregating centres and ultimately, they can carry stress (whereas consonants cannot), around which the phonic profile of words organizes^34^. Therefore, vowels offer an interesting perspective to investigate the workings of the perceptual and motor stages of speech.

Thus, building on previous knowledge on phoneme representation in the brain, we tried to provide a finer characterization of the fronto-temporal language cortex: in fact, we compared modalities of perception, production and articulation imagery within the same pipeline and testing them with a complex vowel model, where all items carry equal complexity. Crucially, we also assessed whether sub-regions within the frontal and temporal hubs of the speech network support high-level, fully phonological representations of vowels exclusively, rather than sharing sensitivity to lower-level acoustic stimuli (pure tones), not pertaining to categorical perception of the salient, linguistic kind.

## Results

### Univariate results

To show regions activated by each of the four tasks, brain activity related to tone perception, vowel listening, imagery and production was contrasted with the resting condition (*p* < 0.05, corrected for False Discovery Rate^35,36^ - FDR), within a topic-based meta-analytic mask of language-sensitive regions selected from the Neurosynth database^37^.

**Figure 1:**
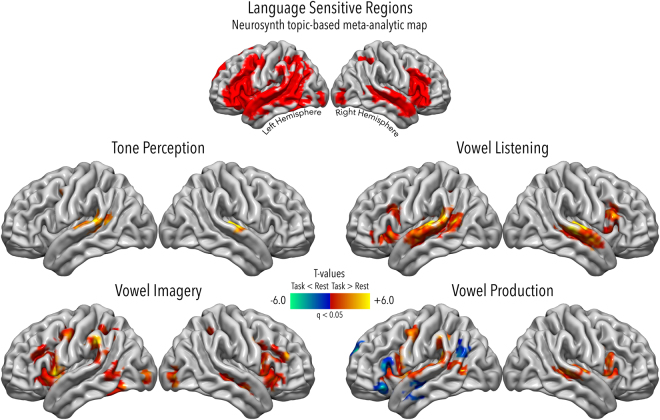
Univariate results. Here the results for one-sample, two-tailed t-tests are shown in each of the four tasks against the resting condition (*p* < 0.05, FDR corrected). These measures were conducted to assess which regions were activated in each task and restricted to a topic-based meta-analytic mask of language-sensitive regions from the Neurosynth database, whose extension can be appreciated in the top panel of this figure.

Figure 1 shows the results of this procedure and the extension of the mask. Particularly, the tone perception task activated the bilateral primary auditory cortex (Heschl’s gyrus, HG) extending to the superior temporal cortex especially in the left hemisphere, along with the superior part of the precentral sulcus (PrCS) at the border with the precentral gyrus (PrCG). In the vowel listening task, HG and superior temporal cortex were activated bilaterally, with more posterior activations in the left hemisphere only; in the frontal cortex, this task activated the left inferior frontal sulcus (IFS) and the opercular portion of the inferior frontal cortex, the insular cortex (INS), and the horizontal ramus of the sylvian fissure, the right *pars opercularis* of the inferior frontal gyrus (IFGpOp), and a small part of the IFS. In the vowel imagery task the frontal cortex was activated in the bilateral (though mostly left) PrCS, left IFS and PrCG, right middle frontal gyrus (MFG/IFS) and bilateral INS; moreover, this task activated significantly the right superior temporal sulcus (STS), left *planum temporale* and supramarginal gyrus (SMG), bilateral intraparietal sulcus (IPS), left posterior middle temporal gyrus (pMTG) and inferior temporal gyrus (ITG), the bilateral middle/inferior occipital gyrus (MOG/IOG), and finally, the bilateral medial portion of the superior frontal gyrus (SFG) and caudate nuclei. The vowel production task significantly activated the bilateral superior temporal cortex extending to the *planum temporale* in the left hemisphere only, the bilateral INS and PrCS, left PrCG, the medial SFG, and left SMG; in this task, significant deactivations were observed in the left hemisphere, particularly in the left *pars orbitalis*, the vertical ramus of the sylvian fissure, the anterior portion of the medial SFG, anterior and posterior portions of the STS.

### Multivariate results

**Figure 2:**
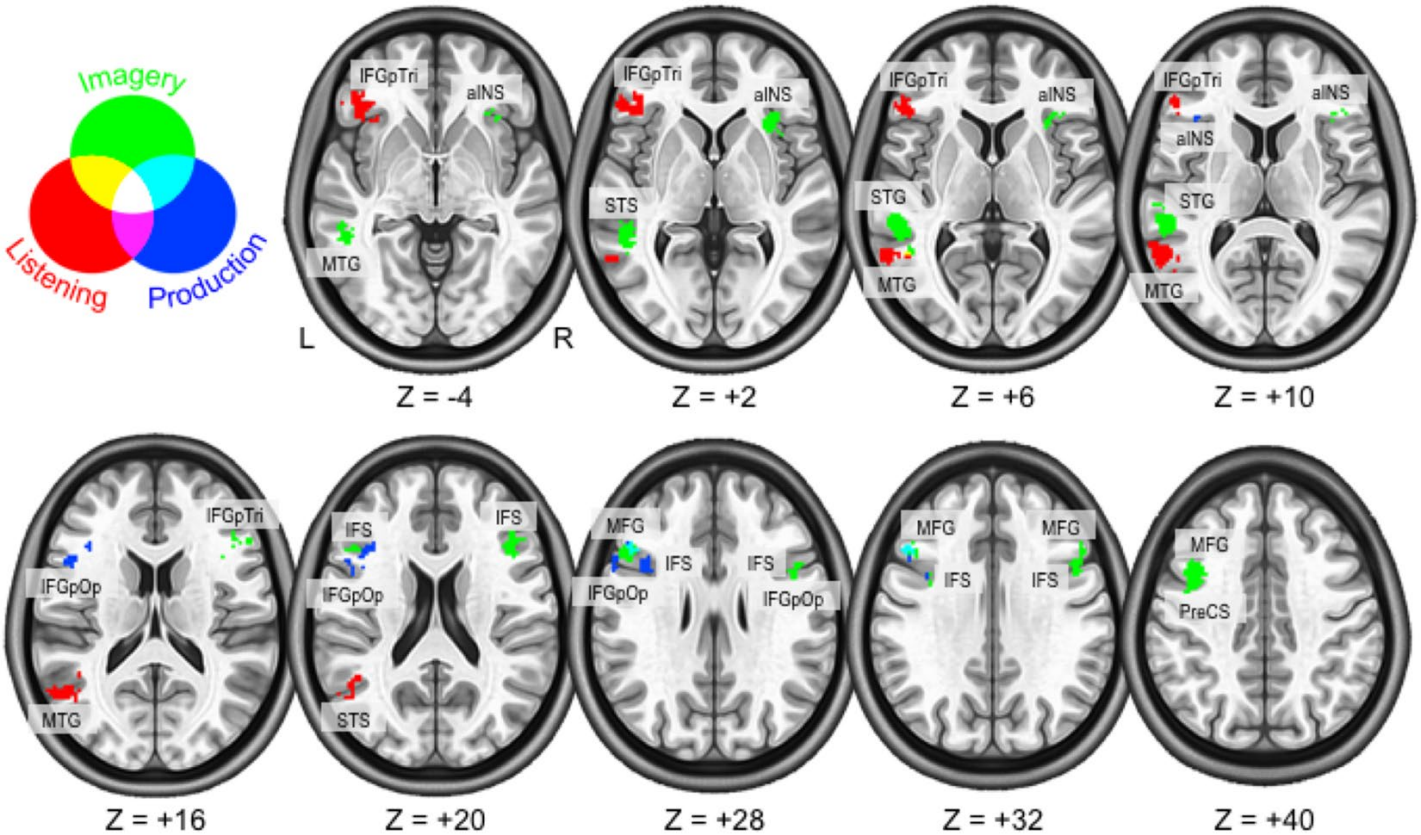
Results mapped on the cortical volume. Here, significant searchlight classifier clusters are shown for the vowel tasks, represented on the cortical volume through axial slices. Colours were assigned by task, and any of their possible combinations were indicated as well in the circle legend. The almost complete contiguity of regions can be appreciated, as marginal overlap emerged only between imagery/production and imagery/listening. No voxels revealed to be shared by all three tasks. Labels are spelled as follows: STS - *superior temporal sulcus*; MTG - *middle temporal gyrus*; IFGpTri - *inferior frontal gyrus, pars triangularis*; STG - *superior temporal gyrus*; IFS - *inferior temporal sulcus*; MFG - *middle frontal gyrus*; IFGpOp - *inferior frontal gyrus, pars opercularis*; aINS - *anterior insular cortex*.

**Figure 3:**
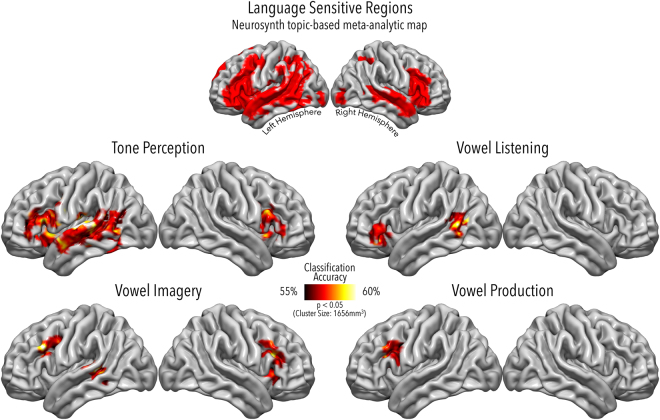
Accuracy maps projected onto the lateral surfaces of the brain. Here we show regions where accuracy values were significant across the searchlight area defined by the selected Neurosynth topic-based meta-analytic map (top panel) in each task (bottom panels). The extension and location of these regions was validated through cluster correction in AFNI at a minimum cluster size of 207 voxels (*p* < 0.05 at voxel level with α < 0.05 for the correction for multiple comparisons).

A multi-class searchlight-based classifier highlighted three sets of clusters, one for each vowel task, where pattern discrimination was successful. Table 1 summarizes the MNI co-ordinates at each cluster’s centre of mass. Figure 2 shows clusters on the cortical volume through axial slices, while Fig. 3 shows the accuracy maps of all experimental tasks projected onto the lateral cortical surfaces.

**Table 1:** MNI co-ordinates and centres of mass for the searchlight-based classifier results.

Task	Cluster	Voxels	CMass x	CMass y	CMass z
Vowel listening	*Left pSTS-MTG*	263	−56.5	−54.8	+11.3
	*Left IFGpTri*	257	−46.5	+32.6	−0.3
Vowel Imagery	*Left pMTG-STG*	352	−53.2	−38.8	+2.5
	*Right IFS-MFG*	346	+42.9	+21.0	+20.0
	*Left IFS-MFG*	230	−44.1	+14.5	+33.7
Vowel Production	*Left IFS-IFGpOp*	211	−45.5	+17.4	+23.9

Here we show the location and extension of clusters emerging from the classifier run within a mask based on language-related studies in the Neurosynth database. For each task, only significant regions are shown that survived cluster correction. Labels are spelled as follows: pSTS - posterior *superior temporal sulcus;* pMTG *- posterior middle temporal gyrus;* IFGpTri *- inferior frontal gyrus, pars triangularis;* STG *- superior temporal gyrus;* IFS *- inferior temporal sulcus;* MFG *- middle frontal gyrus;* IFGpOp *- inferior frontal gyrus, pars opercularis*.

### Vowel listening, imagery and production dissociate in the left inferior frontal cortex

The left inferior frontal cortex (IFG, IFS) was engaged across all experimental conditions, with the addition of the right homologue in the imagery task only. Particularly, though, clusters of voxels within these macro-regions responded specifically to each task (regions were labelled and their overlap with the result masks was interpreted in accordance with the Harvard-Oxford Cortical Atlas). In details, during vowel listening, the *pars triangularis* of the left IFG (IFGpTri) represented vowels, crossing over anteriorly into the *pars orbitalis*. During vowel imagery, the left IFS and its right homologue intersected superiorly the MFG, with a relative overlap with the INS as well. During production, a slightly more posterior region within the left IFS was engaged, running inferiorly into the *pars opercularis* of the IFG, and superiorly into the MFG.

### Vowel listening and imagery dissociate in the superior temporal cortex

Temporal regions representing vowels revealed that the left STG and STS running posteriorly and inferiorly towards MTG, were engaged in listening, as well as performing imagery of vowels through covert articulation. Particularly, temporal regions representing vowels during listening were the left pSTS, extending into the pMTG. Vowel imagery engaged a close-by portion of the left pMTG extending superiorly into the STG and STS. No temporal regions represented vowels significantly during overt production.

### Measuring cross-task spatial segregation and tone sensitivity

No spatial overlap among tasks was revealed, except for a cluster of voxels in the IFS/MFG for vowel imagery and production, and a very small cluster in the MTG for vowel imagery and listening. Moreover, cross-task accuracy measurements revealed that the imagery-sensitive left pMTG-STG region also shared tone representations, as well as IFGpTri during vowel listening. Table 2 summarizes cross-task accuracy results from the calculations performed in each cluster from the vowel tasks, with the associated *p* value and standard errors (SE). Table 3 reports cross-task accuracies for the pure tones within the vowel clusters.

**Table 2:** Cross-task accuracy measures between vowel tasks.

Mask	Task						
	*Cluster*	Vowel listening		Vowel imagery		Vowel production	
		Acc. ± SE	*p*	Acc. ± SE	*p*	Acc. ± SE	*p*
vowel listening	*Left pSTS-MTG*	56.7 ± 1.2	<0.001*	52.8 ± 0.9	0.167	49.8 ± 1	0.958
	*Left IFGpTri*	57.3 ± 0.8	<0.001*	54.1 ± 1.4	0.018	50.3 ± 0.9	0.879
vowel imagery	*Left pMTG-STG*	53.1 ± 1	0.106	57.3 ± 0.9	<0.001*	51.4 ± 0.7	0.645
	*Right IFS-MFG*	52.2 ± 1.2	0.315	56.9 ± 1	<0.001*	52.9 ± 0.9	0.112
	*Left IFS-MFG*	51.7 ± 1.2	0.464	57.1 ± 1.4	<0.001*	53.2 ± 1.4	0.087
vowel production	*Left IFS-IFGpOp*	52.5 ± 1	0.215	53.5 ± 0.9	0.031	56.5 ± 1	<0.001*

Accuracy measures are shown here for each task in its own significant regions, but also compared to the other tasks by constraining the extraction of accuracy values for one task within the areas that were significant in each of the others. Significant values are reported in bold, and gray shading was used to highlight accuracy values within correspondent masks and tasks. Of note, accuracy values were significant only for a task within its own regions, showing no functional overlap between modalities (accuracies were Bonferroni-corrected at *p*_*bonf*_ < 0.0028). For label spelling, please refer to Table 1.

**Table 3:** Cross-task accuracy measures of pure tone perception within each vowel mask.

Mask	*Cluster*	Tone Perception	
		Acc. ± SE	*p*
vowel listening	*Left pSTS-MTG*	53.7 ± 1.2	0.044
	*Left IFGpTri*	54.9 ± 1.5	0.004*
vowel imagery	*Left pMTG-STG*	55.4 ± 1	0.002*
	*Right IFS-MFG*	53.0 ± 1.1	0.112
	*Left IFS-MFG*	51.8 ± 0.8	0.466
vowel production	*Left IFS-IFGpOp*	52.9 ± 1.2	0.134

Tone perception accuracy results were constrained within the masks defined by the vowel classifier. Significant values are reported in bold. Of note, the Left IFGpTri from the vowel listening task and the Left pMTG-STG from the vowel imagery task were also able to represent tones significantly (accuracies were Bonferroni-corrected at *p*_*bonf*_ < 0.0083). For region labels, please refer to Table 1.

## Discussion

In this study, we combined fMRI and MVPA to study the functional organization of vowel listening, imagery and production. We explored the representation of vowels across these three modalities, as well as determining commonalities and differences with a tone perception control task in a frequency range close to that of our speech stimuli. Specifically, patches of cortex in inferior frontal and superior temporal regions retained information to significantly discriminate the seven vowels of the Italian language in each condition. Within these areas, contiguous, and just minimally overlapping clusters were sensitive to listening, articulation imagery and production of speech sounds. Of note, left IFGpTri and left pMTG/STG shared sensitivity to both tones and vowels.

### Functional segregation and tone sensitivity in brain regions involved in vowel listening, imagery and production

Several functional studies explored the representation of vowels, consonants and syllables in the fronto-temporal language areas (although more often considering one task at a time): some highlighted their sensitivity to very fine-grained aspects of speech, such as formant structure, manner and place of articulation, and even speaker identity^7,8,15,38^, while others have highlighted the importance of a shared neural code for validating popular theories about the acquisition and processing of language^17^. Univariate results comparing each of the four tasks (tone perception, vowel listening, imagery and production) against resting condition highlighted a set of regions in line with previous findings, revealing frontal and temporal involvement in language perception and production^1^. However, while classical univariate approaches sought to infer specific mental function by comparing regional average activations, and thus were amply exploited to investigate the spatial organization of speech, multivariate analyses show representational content similarities over regional engagement: this, together with a comprehensive comparison of speech modalities, can provide a finer characterization of the speech function across the fronto-temporal language cortex.

Theoretical approaches seeking to support integration between perception and production have suggested that production and the socially-rooted need for intelligibility in infants can shape perception^2^, so that we refine our produced speech output ever since the babbling phase, just by hearing others’ voices and ours. Alternatively, some have argued that perceived speech would contain articulatory information^3^. In this context, Schwartz and collaborators have tried to reconcile the contrasting ideas that we acquire language by “saying what we should be hearing” or “hearing what we should be saying”, fitting perceptual shaping and motor procedural knowledge together in speech processing^4^. Worth mentioning as well is the functional neuroimaging-based argument of Scott and Johnsrude, suggesting the dual nature of speech as both a sound and an action^39^. In this respect, integration theories argue in favour of a covert articulatory rehearsal mechanism bridging the perception-production gap: such mechanism may be of the utmost relevance in linguistic interactions, whose temporally-fast variations have been frequently associated with the complexity and the computational structure of birdsong, thus integrating functions in sensorimotor learning through efference copies^40^. Importantly, an action-perception dual stream originating in the auditory belt and projecting forward to the inferior frontal cortex, and backward to the parietal lobe, has been proposed for language processes by Rauschecker and Scott^41^, possibly supporting functional integration of the perception-production continuum on the basis of structural connections in humans, and functional studies in the monkey (as well as human) model.

Despite the variety of models proposed, it appears that any theory considering the sharing of neural information between perceived and produced speech should provide an assessment of their *spatial* organization in the frontal and temporal hubs of the speech network. Indeed, a vast amount of literature reveals mixed comprehension and production deficits associated with cortical lesions in these locations^22–25,42–44^, and particularly within the inferior frontal wide territory pertaining to an extended view of Broca’s area^28^, centring around IFGpOp/IFGpTri, touching the lower bank of the PrCG posteriorly and the INS medially. Davis and collaborators, especially, underline that even though a plethora of clinical studies show deficits broadly recollected under the Broca’s aphasia label, not all patients diagnosed with Broca’s aphasia have lesions in the IFGpOp/IFGpTri and not all patients with these kinds of lesions do, in fact, present with all (or some of) Broca’s aphasia-related symptoms^44^; the complexity of lesions and associated disruption of speech along the fronto-temporal network is also reported by Bates and colleagues^45^.

Moreover, recent interest for combining multivariate methods with functional brain data has revealed that phonological information is finely represented in the fronto-temporal language-related cortex: particularly, the superior temporal cortex has been shown to encode perceived phonological features^46^, discrete speech sound categories^7^, and to preserve the representation based on tongue positions together with formant structure^8^. Additional properties have been decoded from perceived phonemes, such as speaker identity, providing an ever-growing account of the complexity of basic speech representations all along the antero-posterior axis of the superior temporal cortex, bilaterally^8,38^.

On the lines of an integrative account, within the prefrontal hub of the speech network, Cheung and colleagues were able to cross-decode *manner* of articulation, a *perceptual* feature of consonants, in *motor* electrodes tested on data previously extracted from the ventral sensorimotor cortex (vSMC) during the production of syllables^17^. Similarly, the involvement of the superior temporal sulcus in processing, at least coarsely, produced syllables was demonstrated, whereas more frontal recordings showed selective firing to specific vowels categories^15^.

Nonetheless, a complete account of the spatial engagement and informational content representation of different speech modalities within the left fronto-temporal cortex is still needed: along these lines, in this study we aimed at extending electrocorticographic findings to the non-invasiveness allowed by fMRI on healthy participants. Notably, while the accuracy and directness of electrocorticography (ECoG) as a measurement of brain function is, indeed, invaluable, fMRI holds the advantage of providing the functional characterization of multiple modalities (perception, production and imagery) across a larger extent of cortex within the same subject, which is not easy to replicate with intracranial recordings, generally tied to clinical needs.

Therefore, to provide a finer spatial and functional account of phonological processing and the production-perception interface, we ran a searchlight classifier of listened, imagined and produced vowels within a mask of neuroimaging studies of the language function. This procedure aimed at measuring the accuracy of vowel discrimination, and, most importantly, the spatial organization and possible overlap between regions controlling the three vowel tasks. Moreover, with the same procedure we attempted tone classification in frequencies close to those of our speech stimuli. Accuracies yielded by each vowel task were also measured in clusters resulting from the classifiers of all the other vowel tasks, as well as tone perception accuracies were tested in the vowel regions.

Globally, our results revealed that speech tasks are indeed processed within two classically linguistic macro-regions in the frontal and temporal cortices. Particularly, though, we did not find production of vowels confined to the inferior frontal cortex, nor perception confined to the superior temporal cortex. Instead, both the inferior frontal and superior temporal cortices represented vowel-specific information in both perception and production (imagined as well as overt). Nonetheless, the three vowel tasks engaged well-defined, *bordering* sub-portions of the inferior frontal and superior temporal hubs, a picture already sustained by lesion studies and pre-operative language function testing^43^. Moreover, the vowel model was well represented in articulation imagery, a task whose aim was to simulate the articulatory rehearsal mechanism assumed by integration theories: even there, segregated regions revealed sensitivity to vowels in contrast with those clusters, adjoining though non-overlapping, which represented perceived and produced stimuli.

Interestingly, though, while no vowel-sensitive regions retained above-chance accuracies for other tasks, two regions represented tones significantly, that is, the IFGpTri involved in listening and the pSTG-MTG involved in imagery of vowels (of note, the region identified in imagery as being tone-sensitive is spatially closer to the primary auditory cortex than the vowel-specific region identified in vowel listening as pSTS-MTG). This result reveals that, while we have regions *within* the frontal and temporal cortices performing both production-related and perception-related functions in a segregated fashion, these areas also retain low-level non-linguistic information. Specifically, though, high-level information pertains only to the “classical” function associated to that area (production in the inferior frontal and perception in the superior temporal cortex), while the “non-classical” associated function is not language-specific (perception in the inferior frontal and articulation imagery in the superior temporal cortex).

Therefore, regardless of how, in fact, we may approach the issue of perception shaping production or *vice versa*, such mechanisms seem to be in place because globally we do not have regions for production or perception of speech *as a whole*. Instead, our findings seem to suggest that the brain retains a capacity for sub-specialization within the classical language fronto-temporal hubs. Speculatively, one may argue that comprehension deficits resulting from lesions within the inferior frontal cortex, as well as production deficits resulting from lesions within the superior temporal cortex, may arise from disruption of lower-level information processing.

### Vowel listening, imagery and production dissociate in the left inferior frontal cortex

Our results showed how vowel listening, as well as vowel imagery and production, engage the left inferior frontal cortex, from the IFGpOp crossing over anteriorly into the IFGpTri, superiorly into the IFS and touching the MFG. Within the right hemisphere, vowel imagery engaged the IFS, MFG and aINS. However, vowel tasks engaged the broad “Broca’s territory” in a functionally segregated fashion: left IFGpOp engaged in vowel production, while the IFS engaged in vowel imagery (as well as its right homologue). Finally, a more anterior region in the IFGpTri engaged in vowel listening although it also represented tones, revealing to be non-specific to speech sounds.

A debate exists on the role of the inferior frontal cortex in processing high- rather than low-level language functions in the healthy brain as well as in lesion studies: this region has been broadly implicated in syntactic working memory^47^, perceptuo-motor integration^48^ and phonetic/phonological representations^19,49^. Furthermore, along the lines of a functional segregation argument, IFGpOp and IFGpTri within Broca’s area have been associated, respectively, to processes pertaining to syntax and semantics^50^. Still, early evidence from Positron Emission Tomography had already suggested that Broca’s area is primed by *any* phonological differences subtending semantic representations, and not by the processing of meaning *per se* ^51^. Moreover, Heim and collaborators do not report additional activations in IFGpTri for semantic *versus* phonological fluency, with only the latter significantly activating IFGpOp^52^.

Along these lines, some have ascribed the disrupted patterns of both complex syntactic *comprehension* and general speech *production* in Broca’s aphasia to a disturbance in the hierarchical chain-processing mechanism at the basis of the phonological loop, which may be controlled by IFGpOp and possibly IFGpTri^44,53^. Recently, it was proposed that Broca’s area in particular mediates the transformation of perceptual information coming first into the superior temporal cortex, thus to be projected back to the PrCG as articulatory instructions for production^54^.

The idea that locations anterior to the PrCG perform sensorimotor transformations and relay information back to the PrCG is in agreement with our findings. Furthermore, we were able to provide a finer characterization of the functional neuroanatomy of the IFG, showing sensitivity to perceived tones and vowels in the *pars triangularis*, and to produced vowels in the *pars opercularis*. Therefore, our results suggest that the language-related inferior frontal cortex, before anything else that may be of a higher level, is concerned *at least* with the representation of perceived speech, as well as non-speech sounds.

The idea that IFGpTri supports simpler, non-linguistic representations, as we found in the cross-task accuracy measurements between vowel listening and tone perception, was previously hinted at by Reiterer and colleagues, who demonstrated IFGpTri involvement in processing tone frequency though not sound pressure, using a pitch *versus* volume discrimination task^55^. On the other hand, Hickok and colleagues reported how IFG-lesioned patients show no *auditory* syllable discrimination deficits whatsoever^23^. Although this result may appear in disagreement with ours, it is reasonable to speculate that the extensions and locations of lesions (as noted by the authors themselves) do not allow for a full comparison with ours and others’ functional results in the healthy brain (as also advised by Ardila and colleagues^25^).

Regarding the *pars opercularis* as the most posterior cluster showing vowel sensitivity, we found produced vowels represented discretely in IFGpOp. In its proximity, the PrCG has been associated to apraxia of speech^42^, a disturbance in the articulatory aspects of production exclusively. Consistently, we were able to discriminate overtly produced vowels at the posterior border of the IFGpOp extending into the PrCS. Instead, vowel imagery involved more anterior regions for the processing of intermediate phonological representations with no sensory output. These arguments appear to sustain the importance of this inferior frontal region at the perceptuo-motor interface for speech.

All in all, our results suggest that both IFGpOp and IFGpTri do perform *phonological* computations, that is, a sub-lexical kind of processing at the basis of any higher-level function (from syntax to semantics, as already mentioned), and their spatial organization is rather driven by the speech task being performed, with perception and production completely detached, and perception being non-specific to speech sounds.

In fact, some of those trying to reconcile the vast literature on inferior frontal cortex involvement in speech processing have argued that, if its engagement is a matter of perceptuo-motor interface, then the IFG *as a whole* should share activations related to different tasks in the speech loop^56^. This argument has been brought forward particularly by those sustaining that region sharing would constitute a neurofunctional correlate of mainframes such as the MTSP^3^. Our results, instead, reveal functional *dissociation* within the inferior frontal cortex for different tasks related to speech sound discrimination, and clarify at least the correlation of *both* IFGpOp and IFGpTri with phonological-level functions.

The processing of produced and imagined speech in close-by regions, as well as more anterior and more rightward activations for imagined speech, were previously reported^57,58^. In our results, we found a cluster of spatial overlap between the regions involved in produced and imagined vowels in the IFS/MFG. This location’s centre of mass was associated to cognitive processes related to working memory in the Neurosynth database (highest posterior probability: ‘retrieved’ 0.77, ‘memory retrieval’ 0.76, ‘wm task’ 0.76). Of note, our subjects were asked to maintain and then retrieve a heard vowel thus to perform imagery or production, and the searchlight analysis was then conducted on the retrieval phase of the trials. In this sense, the small cluster of spatial overlap that we found between production and imagery could be explained as a common *focus* for the mnemonic-attentive component of the task (vowel retrieval). To reinforce this argument, cross-task accuracy measurements did not reveal shared sensitivity to produced *and* imagined vowels in this region, instead showing complete dissociation: in fact, that cluster of spatial overlap may be shared by the production and imagery-sensitive clusters for task-specific demands, and not information content representation.

Finally, the involvement of the right IFS-MFG homologue, as well as aINS, in the imagery task would be justifiable in that these regions were shown to be involved in mental/imagined speech^59^ and aphasia recovery in left IFG/IFS-lesioned patients^57,60^.

### Vowel listening and imagery dissociate in the superior temporal cortex

In our study, the left superior and middle temporal cortices were largely engaged by vowel listening and vowel imagery. Regarding the engagement of the superior temporal cortex in perceived speech, a large body of evidence suggests that this region retains sensitivity to complex harmonic structures and, generally, spectral features down to a stimulus-specific level, studied with both fMRI^8,38^ and ECoG^7,46,61^. The superior temporal cortex has been associated also to imagery of speech, arguing that the pSTG-pSTS-MTG macro-region supports *both* imagery and perception^62,63^. Interestingly, though, our results showed that vowel listening and vowel imagery dissociate spatially, as in the inferior frontal cortex; moreover, pSTG-MTG retains tone-specific representations as well as imagined vowels. This reveals how, in the superior temporal cortex as well as the inferior frontal, the function classically associated to the region is language-specific, while the non-classical function shares sensitivity to lower-level stimuli.

Among those who argued in favour of an integrated model, Murakami and colleagues^64^ found that repetitive transcranial magnetic stimulation over the left superior temporal cortex can disrupt phonological fluency, in that it suppresses muscular evoked potential facilitation in the primary motor cortex. This evidence may be of help in characterizing our vowel imagery result in left pSTS-MTG, in that it may validate the idea that mechanisms springing from inferior frontal, speech-generating areas modulate activity in speech-perceiving ones, during covert articulation^65^. It is worth mentioning again that vowels arise from a perceptuo-motor model, with formant structure being determined by unique articulator configurations^34^. Such a model would contain both acoustic and motor information, and thus be represented equally well in superior temporal and inferior frontal areas. These findings are in agreement with previous results obtained with MVPA on functional brain imaging^8^ as well as ECoG data^7^ showing not only that the auditory cortex can encode vowel-specific information during perception^7^, but also, that it can represent articulated speech sounds^15^. Particularly, though, HG, the primary auditory cortex, did not show sensitivity to single phonemes^8^, as our findings confirm, despite the exquisitely acoustic nature of the task. Nonetheless, in our univariate results HG was significantly activated during vowel listening (see Fig. 1), although it represented pure tones in the multivariate results (see Fig. 3): an extrapolation coming from MVPA is that HG was simply not representing vowels in the listening task, despite being activated, as can be seen from Fig. 1. Of note, as explained in the Methods section, vowels are aggregates of formants above a fundamental frequency, which are perceived as a summation of the fundamental and the overtones, but also as discrete categories^7^. Such kind of complex stimuli with heightened (linguistic) salience might be computed outside the psychophysically low-level HG^66,67^, as our findings seem to suggest in comparison with simpler tones that are, indeed, represented there. Finally, findings from task-dependent decoding of speaker and vowel identity^38^ reveal that the primary auditory cortex in the left hemisphere actually represents speaker information over vowel information, which seems reasonable when we consider the higher frequential variability of different speakers (across which is the fundamental frequency that changes), rather than the small changes in different vowels uttered by the same speaker, related to harmonic structure over the same fundamental^34^.

Moreover, in Tankus and colleagues^15^, while STG was further probed to assess its ability to discriminate between a complex system of five vowels, the authors also showed how this classically auditory hub of the cortex actually represents *articulated* speech sounds as well: nevertheless, while neurons in anterior locations such as the medial orbitofrontal cortex (MOF) and the rostral anterior cingulate cortex (rAC) responded to single or coupled vowels, in this study STG did not, in fact, reveal vowel specificity. In agreement with this study, we found STG activated by vowel production (Fig. 1), but crucially it did not classify single vowels (Fig. 3).

Moreover, pSTS-MTG, previously shown to be engaged in articulation imagery over *hearing* imagery^32^, shared sensitivity to mentally articulated vowels, as well as pure tones, in our data: this is supported by a study reporting conflict between vowel imagery and tone perception in the superior temporal cortex^68^. As in our findings, the region showing shared sensitivity to lower- and higher-level stimuli was significantly lateralized in the left, language-dominant hemisphere. Moreover, in our results, the patterns of imagined vowels that were represented in left pSTS-MTG could not be ascribed to any acoustic feedback due to the inner nature of the task itself. In this region, tone sensitivity would therefore sustain higher-level representations pertaining to a non-classical function associated to the location, as well as it did in the inferior frontal cortex.

In conclusion, using fMRI we were able to discriminate the seven vowels of the Italian language in listening, articulation imagery, and production tasks. Globally, these three functions revealed spatial dissociation within language-related brain regions, as well as collateral sensitivity to tone representations. Building on previous evidence, and on suggestions coming from theories postulating the integration of the perceptual and articulatory stages of speech, these findings provide a finer characterisation of the fronto-temporal language-related cortex. Notably, frontal brain regions classically associated to production can also represent acoustic features of both linguistic and non-linguistic stimuli; similarly, temporal regions that process low-level acoustic features (pure tones) retain sensitivity to covertly produced vowels. Importantly, in line with integration theories, not only sensitivity to speech listening exists in production-related regions and *vice versa*, but the nature of such interwoven organisation is also built upon low-level perceptual features.

## Methods

### Participants

Fifteen right-handed (Edinburgh Handedness Inventory^69^, mean laterality index 0.79 ± 0.17) healthy, mother-tongue Italian monolingual speakers (9 F; mean age 28.5±4.6 years) participated in this study, after its approval by the Ethics Committee of the University of Pisa. All experimental procedures and methodologies were carried out in accordance with the relevant guidelines and regulations. Informed consent was gathered from all participants.

### Stimuli

**Figure 4:**
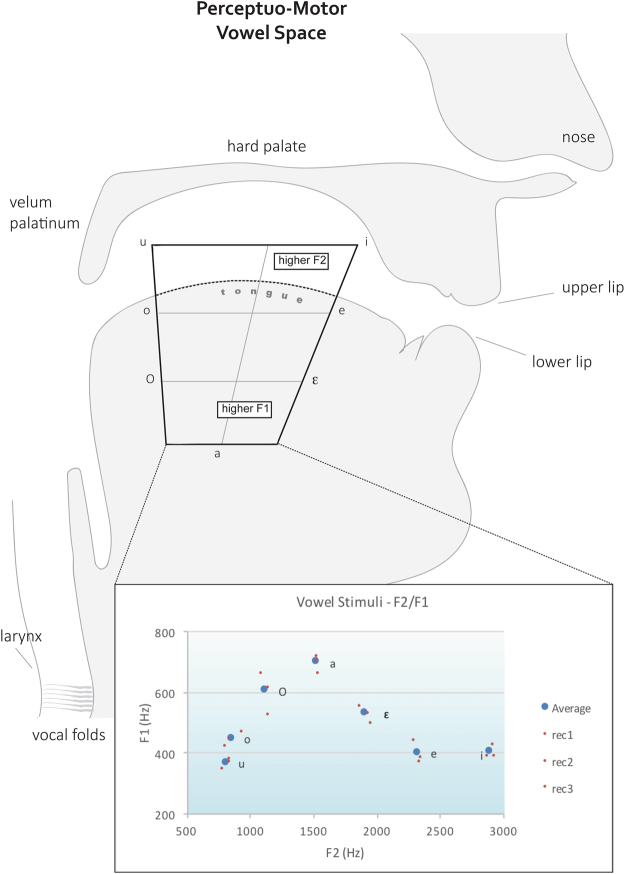
Vowel acoustic and motor spaces. Here, an ideal representation of the perceptuo-motor vowel space can be appreciated through a sagittal view of the head and phonatory apparatus (top). The articulators are labelled and the relationship that lip and tongue positions entertain with the first and second formant (F1 and F2) can be seen from the trapezoid shape representing the Italian vowel system. Below, the real first- and second formant measurements from our experimental stimuli are plotted in the F1/F2 space, reproducing a projection of the pictured perceptuo-motor vowel space. In this chart, averages for each vowel are represented with blue dots, while measures from single recordings are represented with smaller, red dots (see legend: rec - recording).

The seven vowels of the Italian language ([i] [e] [ε] [a] [ɔ] [o] [u]) were selected as experimental stimuli, along with seven pure tones (450, 840, 1370, 1850, 2150, 2500, 2900 Hz). Pure tones are physically simpler sounds with no harmonic structure, whereas vowels, despite being periodic waves as well, are endowed with acoustic resonances at specific frequency bandwidths, determined by the vocal tract modifying the source signal produced by the laryngeal mechanism. This structure yields a continuous emission of sound with a fundamental frequency (F0) and a number of overtones called *formants* (i.e., F1, F2, F3…), in a combination that is unique for each vowel. The seven vowels from the Italian phonemic inventory can be disambiguated by the two lower formants F1 and F2, with F0 being constant (Fig. 4)^34^.

Three separate, 2 s natural voice recordings of each vowel (21 stimuli) were obtained from a female Italian speaker using Praat (©Paul Boersma and David Weenink, http://www.fon.hum.uva.nl/praat/) a 44100 Hz frequency sampling rate (F0: 191 ± 2.3 Hz) and spectrograms were visually inspected for abnormalities. Pure tones were selected by dividing the minimum/maximum mean F1 range of the vowel set into seven, equally distanced bins; the resulting values were approximated to the closest Bark scale value and then converted back to Hertz, so that all tones would lie within the sensitive perceptual bands in a psychophysical model^70^. In Audacity (©Audacity Team, http://audacity.sourceforge.net/), seven tones were thus generated using the input-frequencies associated to the Bark value obtained through the aforementioned procedure. Table 4 reports mean F1 and F2 across recordings with the associated standard deviations, and the resulting approximated Bark value from which pure tones were generated.

**Table 4:** Mean F1 and F2 across recordings for the vowel stimuli.

Vowels	Mean F_1_	STD	Mean F_2_	STD	Bark Value	Tones
**i**	305	21.1	2170	25.7	5	**450**
**e**	303	35.9	1736	30.7	8	**840**
**ɛ**	400	27.1	1428	47.4	11	**1370**
**a**	525	28.9	1139	7.1	13	**1850**
**ɔ**	455	68.1	836	34.9	14	**2150**
**o**	338	23.4	637	71.6	15	**2500**
**u**	278	16.2	604	27.0	16	**2900**

Here we show the Bark scale -to-Hertz conversion for the pure tones used in the tone perception task. Tone frequencies were obtained starting from mean vowel frequencies (left-to-right in the table). All vowels and tones lasted for 2 seconds.

### Experimental procedures

A slow event-related paradigm was implemented with Presentation (©Neurobehavioral Systems, Inc., http://www.neurobs.com/) and comprised two perceptual tasks (tone perception and vowel listening), a vowel imagery task and a vowel production one. To increase the amplitude of individual BOLD responses during scan time, all perceived vowels and tones, as well as the execution of imagery and production, were made to last for 2 whole seconds, with the duration signalled by a green fixation cross that would turn black during resting time. All perceptual stimuli (tones or vowels) were thus administered in trials comprising 2 s stimulus presentation, then followed by 8 s rest. Imagery/production stimuli were administered in trials comprising 2 s stimulus presentation, 8 s maintenance, 2 s task execution and 8 s rest. For the imagery task, participants were instructed to perform mental articulation of a heard vowel with their own voice and simulating speech in their mind without ever moving; for the production task, they were instructed to speak naturally and at a normal volume, with rubber wedges and pillows secured so as to avoid head motion without constraining the chin and jaw. In the perceptual tasks (tone perception and vowel listening) subjects were instructed to lay still and listen attentively to the presented stimuli. Globally, functional scans were 47 m long, divided in 10 runs. Each of the three vowel recordings was presented twice, thus to obtain 42 trials randomized within and across tasks and subjects, with each sound, either vowel or tone, being equally represented.

BOLD activity was measured using GRE-EPI sequences on a GE Signa 3 Tesla scanner (TR/TE = 2500/30 ms; FA = 75°; 2 mm isovoxel; geometry: 128 × 128 × 37 axial slices). Brain anatomy was provided by a T1-weighted FSPGR sequence (TR/TE = 8.16/3.18 ms; FA = 12°; 1 mm isovoxel; geometry: 256 × 256 × 170 axial slices). Stimuli were presented using MR-compatible on-ear headphones (30 dB noise-attenuation, 40 Hz to 40 kHz frequency response).

### fMRI pre-processing

The AFNI software package^71^ was used to pre-process functional MRI data. First, all acquired slices were temporally aligned within each volume (*3dTshift*), corrected for head motion (*3dvolreg*), spatially smoothed (*3dmerge*) with a 4 mm FWHM Gaussian filter, and normalized by dividing, within each voxel, every time point by the mean of the time series. A multiple regression analysis was then performed on normalized runs (*3dDeconvolve*), to identify stimulus-related BOLD patterns. Movement parameters and signal trends were included in this procedure as regressors of no interest. Specifically, we used TENT functions for the estimation of BOLD activity (T-values), focusing on the third time point (7.5 seconds) after the acoustic stimulus onset or task execution (imagery or production). By doing this, we aimed at limiting sensory-motor and maintenance-related information, possibly biasing the signal preceding vowel imagery and production^72–74^. BOLD activity related to the acoustic stimulation in the imagery and production tasks was discarded. Afterwards, T1 images were pre-processed in FSL^75^ and nonlinearly registered^76^ to the Montreal Neurological Institute (MNI) standard space with a 2 mm isovoxel^77^; then, the obtained deformation field was used to warp functional maps for each task type.

### Language-sensitive regions

Hereon, all analyses were performed within a pre-defined topic-based meta-analytic mask of language-sensitive regions. Specifically, the mask was selected from the Neurosynth database^37^, version 3, topic 21 out of 200, forward inference with a *p* < 0.01 (FDR corrected)^78^. Keywords included terms related to language and phonological competence, among which “speech, auditory, sounds, processing, perception, voice, pitch, listening, production, vocal, tones, voices, phonetic, syllable, linguistic, speaker, discrimination, spectral, vowel, language”. The extension of the mask was 19093 voxels and comprised the bilateral posterior portion of the IFG/MFG, the left PrCG, the bilateral superior temporal cortex, running more posteriorly in the left hemisphere; the left ITG, SMG and angular gyrus (AG), and the bilateral IPS and MOG/IOG. The mask also included the bilateral caudate nuclei, and the medial portion of the SFG. All analyses, both univariate and multivariate, were performed within this mask.

### Univariate Analysis

BOLD activity was used to perform one-sample 2-tailed t-test voxel-wise (*p* < 0.05, FDR corrected), thus comparing task activity versus rest in each modality.

### Multivariate Analysis

To assess stimulus discrimination accuracy in each task, the T-value maps were then used in four searchlight-based classifiers^79,80^ (rank accuracy; cosine similarity; 6 mm searchlight radius), one for each task (tone perception, vowel listening, imagery and production). A cross-validation *leave-one-stimulus-out* procedure was adopted to measure classification accuracy.

Each classifier was conceived to discriminate among seven classes of stimuli: the seven tones in the tone perception task and the seven vowels in the listening, imagery and production tasks. Accuracies emerging from the tone perception classifier would be used later on, to measure sensitivity to low-level features of acoustic stimuli within clusters defined by the vowel classifiers. Finally, the procedure generated a stimulus discrimination accuracy value for each task, in each voxel and subject. Group accuracies for tone perception, vowel listening, imagery and production were obtained by averaging all single-subject accuracy values, at each voxel.

To assess significance, group accuracies were tested against chance by a permutation test^81–83^, where all stimulus-class labels were shuffled in order to generate 1,000 permuted matrices to be used in a multi-class searchlight-based classifier identical to the one described above. The entire procedure generated a set of 1,000 single-subject null discrimination accuracies for each stimulus class, in each voxel, subject and task. Group null accuracies were obtained by averaging single-subject null accuracies in a distribution of 1,000 null accuracies for each voxel and stimulus class. Group accuracy maps were then corrected for multiple comparisons using AFNI: first, real smoothness in the data (resulting from pre-processing, anatomical and searchlight-related smoothing) was estimated (*3dFWHMx*) from the null distribution defined above; later, cluster correction was performed using Monte Carlo simulations (the latest version of *3dClustSim*, 10,000 iterations^84^). This procedure preserved clusters larger than 207 voxels (*p* < 0.05 at voxel level with *α* < 0.05 for the correction for multiple comparisons). All the procedures were developed in Matlab (©TheMathWorks, Inc., http://www.mathworks.com/), unless otherwise specified, through code developed in-house.

### Cross-task accuracies

To assess whether vowel-sensitive clusters were specific to each task, we measured the averaged accuracies of each task within the masks defined by each of the others (e.g., accuracy of vowel listening within the vowel production mask; *3dROIstat*s). The same procedure was applied to the null distribution used in the aforementioned permutation test, thus to obtain cluster-based accuracies and their associated statistical significance (1,000 permutations, one-tailed rank test, *p* < 0.05). Finally, significance level was adjusted using Bonferroni’s correction for multiple comparisons (6 clusters by 3 tasks, *p* < 0.0028 for *p*_*bonf*_ < 0.05). The same procedure was employed to assess whether vowel-sensitive clusters represented tone-related information as well, thus to assess their specificity to non-linguistic versus linguistic stimuli; results were Bonferroni-corrected as well (6 clusters by 1 task, *p* < 0.0083 for *p*_*bonf*_ < 0.05).

### Data availability

The datasets generated and analysed during the current study are available from the corresponding author on reasonable request.
